# Inducible clindamycin resistance in *Staphylococcus aureus* isolates recovered from Mashhad, Iran

**Published:** 2012-06

**Authors:** N Seifi, N Kahani, E Askari, S Mahdipour, Nasab M Naderi

**Affiliations:** 1 Mashhad Medical Microbiology Student Research Group, Mashhad University of Medical Sciences, Mashhad; 2 Department of Medical Bacteriology and Virology, Imam Reza Hospital, Faculty of Medicine, Mashhad University of Medical Sciences, Mashhad, Iran

**Keywords:** *Staphylococcus aureus*, clindamycin, Inducible resistance

## Abstract

**Background and Objectives:**

*Staphylococcus aureus* is an important agent in hospital and community-associated infections, causing high morbidity and mortality. Introduction of the new antimicrobial classes for this pathogen has been usually followed by the emergence of resistant strains through multiple mechanisms. For instance, resistance to clindamycin (CLI)can be constitutive or inducible. Inducible clindamycin resistance which may lead to treatment failure can simply be identified by performing D-test. The aim of this study was to determine the prevalence of inducible clindamycin resistance among *Staphylococcus aureus* isolates by D-test method.

**Materials and Methods:**

This was a cross-sectional study conducted on 211 non-duplicated *S. aureus* isolates in Imam Reza hospital of Mashhad during 2010. Susceptibility to oxacillin, cefoxitin, erythromycin and clindamycin was performed by agar disk diffusion method according to CLSI guidelines and D-shaped clindamycin susceptibility patterns where considered as D-test positive (D^+^).

**Results:**

Of 211 *S. aureus* isolates,88 (41.7%) were methicillin resistant. It was found that of 88 MRSA isolates, 78 (88.6%) were erythromycin (ERY) resistant and 46 (52.3%) were CLI resistant. ERY and CLI resistance in MSSA strains was 22% and 10.6% respectively. Inducible clindamycin resistance was detected in 18 (20.5%) MRSA isolates. 47(53.4%) of MRSA isolates and 9 (7.3%) of MSSA showed constitutive MLS_B_ phenotype.

**Conclusion:**

In conclusion, we found a high prevalence of inducible clindamycin resistance phenotype in our region. We recommend that whenever clindamycin is intended to be used for *S. aureus* infections, D-test should be performed to facilitate the appropriate treatment of patients.

## INTRODUCTION

*Staphylococcus aureus* (*S. aureus*) is one of the most common organisms causing nosocomial and community-acquired infections worldwide (1–3). About 30% of the general population is colonized with *S. aureus* and up to 3% carry methicillin-resistant *Staphylococcus aureus* (MRSA) in their nose (4). These bacteria can cause a wide range of infections from mild folliculitis to potentially fatal systemic illnesses such as bacteremia or endocarditis (5). The increasing prevalence of methicillin resistance among staphylococci is an increasing problem. In England and Wales, during 2006-10, 0.2% of all deaths and 0.4% of hospital deaths were attributed to MRSA (4). Nasal carrier individuals may develop many clinical infections. Despite limited consequences in extramural settings, it has been demonstrated that in certain groups of patients (e.g., those undergoing surgery or hemodialysis and HIV-positive patients), nasal carriage of *S. aureus* plays an important role in the development of infection (6, 7). Treatment of MRSA strains often require different types of antibiotics (3, 4) and this makes it more difficult to treat staphylococcal infections (8).

Macrolide, lincosamide, streptogramin (MLS_B_) antibiotics are commonly used in treatment of staphylococcal infections. In this group, clindamycin (CLI), with its excellent pharmacokinetic properties, is a common choice to treat skin and soft tissue infections (1, 2, 9). Its efficacy in the treatment of respiratory tract, bone and joint infections has also been confirmed. With the low incidence of gastrointestinal side-effects, it is suitable for prolonged therapy. It is also an alternative in penicillin-hypersensitive patients, and an important therapeutic option in outpatient therapy or as follow-up after intravenous therapy (2). It has also been indicated to inhibit the production of *S. aureus* toxins (10, 2).However, widespread use of MLS_B_ antibiotics and unrestricted macrolide usage in Iran has led to an increase in the prevalence of staphylococcal strains which develop resistance to these antibiotics (7, 11).

Macrolide and lincosamide resistance is mainly due to one of these three mechanisms (12):

Target site modification: Ribosomal methylation or mutation which prevents binding of antibiotic to its ribosomal target. This is the most prevalent mechanism of resistance to macrolides and lincosamides encoded by *erm* genes.

Efflux of antibiotic: encoded by *msrA* gene

Drug inactivation**:** encoded by *lnu* genes

Modification of ribosomal target which confers broad-spectrum resistance to macrolides and lincosamides, is encoded by a variety of *erm* (erythromycin ribosome methylase) genes. *ErmA* and *ermC* are typically staphylococcal genes. This mechanism can be constitutive (cMLS_B_); always producing the rRNAmethylase, or inducible (iMLS_B_), that is producing methylase only in the presence of an inductor (2).

It has been demonstrated that clindamycin treatment in patients with iMLS_B_ may lead to cMLS_B_ and therapeutic failure (13). The best way to detect inducible clindamycin resistance (ICR) is a test known as disk approximation test or D-test.

Frequencies of different resistance phenotypes vary by hospital and geographical regions, patient group, bacterial strains and bacterial susceptibility pattern (10). The aim of the present study was to determine the percentage of *Staphylococcus aureus* isolates having inducible clindamycin resistance in our geographical area using D-test.

## MATERIALS AND METHODS

In this cross-sectional study which was conducted, a total of 211 *S. aureus* isolates were collected from Imam Reza Hospital (IRH) in Mashhad during 2010. Duplicate isolates from the same patient were not included in the study. Isolates were obtained from different wards. Most of them were from pediatrics, burns, internal medicine, infections and tropical diseases, and emergency departments.

The isolates were first identified as *S. aureus* by standard biochemical techniques and conventional methods (colony morphology, Gram stain, catalase activity, and slide and tube coagulase test).

The isolates were tested for susceptibility to clindamysin (2µg) and erythromycin (15µg) (Mast, UK). To detect MRSA isolates we used oxacillin (1 µg) and cefoxitin (30 µg) disks (Mast, UK). An inhibition zone of 10mm or less around oxacillin disk indicates MRSA. In regards of cefoxitin disk, inhibition zone of less or equal to 21mm was indicated as MRSA. Isolates that were CLI susceptible and erythromycin resistant (ER-R) were tested for inducible resistance by the use of D-test.

**Fig. 1:**
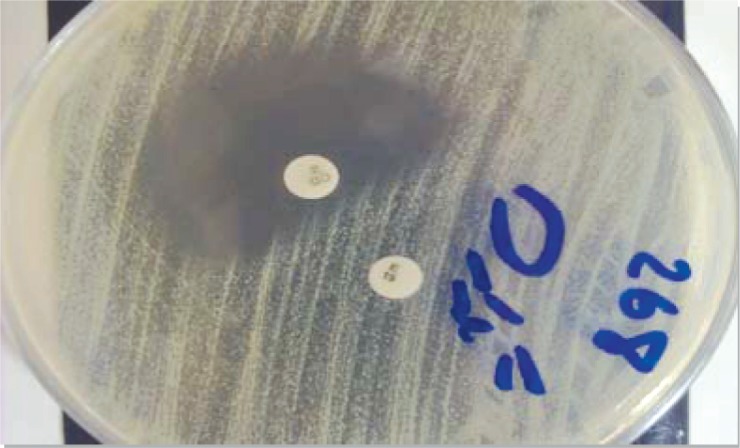
D-zone of inhibition around clindamycin disk indicates the inducible MLS_B_ phenotype.

A 0.5 McFarland equivalent suspension of organisms was incubated on Muller-Hinton agar (MHA) plate as described in the CLSI recommendations (Clinical Laboratory Standard Institute, 2009) (14). Clindamycin and erythromycin disks were placed 15-26mm apart from each other on the MHA plates. After 18h incubation at 37^○^C, plates were checked. Flattening of inhibition zone (D-shaped) around clindamycin was considered as inducible clindamycin resistance (Fig. 1).

The test allows for identification of four different phenotypes:

The inducible MLS_B_ phenotype (D^+^): Resistant to erythromycin and susceptible to clindamycin with a D-zone of inhibition around the clindamycin disk.

The constitutive MLS_B_ phenotype: Resistant to both erythromycin and clindamycin.

The MS_B_ phenotype: Resistant to erythromycin and susceptible to clindamycin.

The susceptible phenotype: Susceptible to both clindamycin and erythromycin.

Data were analyzed by SPSS (ver. 16.0). Chi square test was applied for statistical analysis and level of significance was considered as 0.05.

## RESULTS

Of 211 *S. aureus* isolates, 140 (66.4%) were recovered from male patients and 71 from females. The average age of male and female patients was 38.8± 26.1 and 34.7± 26.4 respectively. (Total: 37.4± 26.2).

**Fig. 2:**
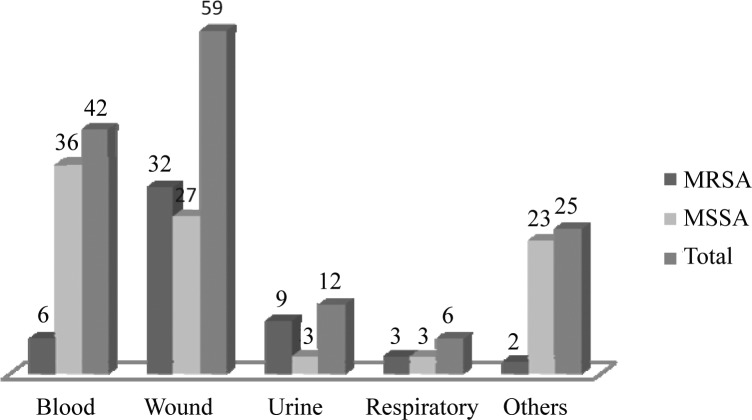
Distribution of MRSA and MSSA strains according to their source of recovery.

Eighty-eight isolates (41.7%) were MRSA. Among 144 specimens with available demographic data, blood and wound infections accounted for the most prevalent specimens collected from inpatients (Fig. 2).

It was found that out of 88 MRSA isolates, 78 (88.6%) were erythromycin (ERY) resistant and 46 (52.3%) were CLI resistant. ERY and CLI resistance in MSSA strains was 22% and 11.4% respectively. (Table 1)

**Table 1:** Antibiotic susceptibility of MRSA and MSSA isolates.

	MRSA (n = 88)				MSSA (n = 123)			
	S	I	R	D	S	I	R	D
Erythromycin	9	1	78	–	92	4	27	–
Clindamycin	23	1	46	18	94	9	13	7

MRSA = Methicillin Resistant *Staphylococcus aureus*; MSSA = Methicillin Susceptible *Staphylococcus aureus*; S = susceptible; I = intermediate; R = resistant

Inducible clindamycin resistance was detected in 18 (20.5%) MRSA isolates. Forty-six (52.3%) MRSA isolates and nine (7.3%) MSSA showed constitutive MLS_B_ phenotype. (Table 2)

**Table 2:** Susceptibility of *S. aureus* strains to erythromycin and clindamycin.

	MRSA		MSSA		Total		P- value
	
	(n = 88)	(%)	(n = 123)	(%)	(n)	(%)	
ER-S, CL-S	9	10.22	91	73.98	100	47.39	
ER-R, CL-R	47	53.40	9	7.32	56	26.54	<0.001
ER-S, CL-R	1	1.13	5	4.06	6	2.84	<0.001
ER-R, CL-S (D^−^)	13	14.77	12	9.76	25	11.85	<0.001
ER-R, CL-S, (D^+^)	18	20.45	6	4.88	24	11.37	0.17
							<0.001
Total (%)	88	100	123	100	211	100	

MRSA = Methicillin Resistant *Staphylococcus aureus*; MSSA = Methicillin Susceptible *Staphylococcus aureus*; E =erythromycin; CL = clindamycin; S = susceptible; R = resistant; D^−^=D-test negative; D^+^=D-test positive

## DISCUSSION

MRSA is now one of the most common nosocomial pathogens in many countries. Early detection of MRSA and formulation of effective antibiotic policy is of high importance (15). In our study, 41.7% of examined isolates were found to be methicillin resistant. In 2009, a similar prevalence of 41% was reported from Tabriz (16). Ekrami reported prevalence of 60% in 2011 from Ahvaz (17). In India (2011) similar prevalence was reported in different studies. (29.1%, 27.97%, 26%) (1, 10, 14). Nearly the same result (26%) was published from Turkey in the same year (18). The prevalence of MRSA reported from US was 55.7% among inpatients and 48.7% among outpatients (19). The different MRSA prevalence reported from different countries suggests targeted surveillance to obtain local resistance data which can lead to the most effective therapy considering all consequences long term (20).

Frequencies of different resistance phenotypes vary by hospital and geographical regions, patient group, bacterial strains and bacterial susceptibility pattern (10). In the present study, the prevalence of iMLS_B_, cMLS_B_ and MS_B_ resistance phenotype was 11.37%, 26.07% and 12.32% respectively. In a previous report from our hospital, 0.7% of methicilin resistant staphylococci isolates represent the iMLS_B_ phenotype (21). In a recent study from Iran, 6.4% of isolates had the iMLS_B_ phenotype and 92.8% were constitutively resistant. The MS_B_ phenotype was only seen in 0.8% of isolates (22). Memariani reports a higher incidence of iMLS_B_ phenotype from Iran (20.7%) (23). However, in some other studies from Iran, the reported incidence is lower (9.7%, 5.2% and 5.3%) (2, 24, 25). In Texas, Chavez-Bueno reported the decreasing incidence of iMLS_B_ phenotype from 1999 to 2002. The prevalence of D^+^ isolates among CA-MRSA was reported to be 93% in 1999; 64% in 2000; 23% in 2001 and 7% in 2002 (26).

The difference observed between the prevalence of inducible and constitutive MLS_B_ resistance was demonstrated to be statistically significant in MRSA and MSSA isolates (p < 0.001). In Turkey, the prevalence of iMLS_B_, cMLS_B,_ and MS_B_ phenotype among MRSA strains was 18%, 23%, 48% respectively. Lower prevalence was reported from MSSA strains. (2%, 3%, 16% respectively) (18). While in our study 20.45% of MRSA isolates had iMLS_B_ resistance phenotype, and the prevalence of cMLS_B_ and MS_B_ resistance phenotypes was 52.27 and 15.91 percent respectively. Saderi from Iran reported no MS_B_ phenotype among MRSA isolates in 2009, though 9.3% of isolates had inducible and 83.9% had constitutive MLS_B_ phenotype (2). A total prevalence of 10.52% was reported from India in 2011 for iMLS_B_ resistance phenotype. (20% in MRSA and 6.15% in MSSA isolates) (1). In Canada, the prevalence of inducible and constitutive clindamycin resistance among MRSA isolates was 64.7% and 35.3% respectively and in MSSA group it was 90.4% and 8.5% respectively (27). The prevalence of iMLS_B_ phenotype among MSSA isolates in our study was 4.88%, much lower than what was reported from Canada. In our study, the level of constitutive clindamycin resistace among MSSA isolates was 7.32%. In Libya it was 9.1% and in Illinois 1-2% (10, 28).

Higher prevalence of iMLS_B_ phenotype in MRSA infections compared to MSSA infections suggests that clindamycin therapy for MSSA infections is successful in many circumstances while it may lead to treatment failure for MRSA infections.

In conclusion, we found a high prevalence of inducible clindamycin resistance phenotype in our region, and it is considerably higher than our previous report (21). We recommend that whenever clindamycin is intended to be used for *S. aureus* infections, D-test should be performed to facilitate the appropriate treatment of patients.
